# The iratebirds Citizen Science Project: a Dataset on Birds’ Visual Aesthetic Attractiveness to Humans

**DOI:** 10.1038/s41597-023-02169-0

**Published:** 2023-05-19

**Authors:** Anna Haukka, Aleksi Lehikoinen, Stefano Mammola, William Morris, Andrea Santangeli

**Affiliations:** 1 The Helsinki Lab of Ornithology, Zoology Unit, The Finnish Museum of Natural History (LUOMUS), University of Helsinki, Helsinki, Finland; 2 Molecular Ecology Group (MEG), Water Research Institute (IRSA), National Research Council (CNR), Verbania Pallanza, Verbania, Italy; 3 Laboratory for Integrative Biodiversity Research (LIBRe), Finnish Museum of Natural History (LUOMUS), University of Helsinki, Helsinki, Finland; 4 The Finnish Museum of Natural History (LUOMUS), University of Helsinki, Helsinki, Finland; 5 Population Ecology Group, Institute for Mediterranean Studies (IMEDEA), CSIC-UIB, 07190 Esporles, Spain

**Keywords:** Conservation biology, Zoology

## Abstract

Amidst a global biodiversity crisis, shedding light on the factors that make us like a species can help us understand human’s nature-related attitudes and inform conservation actions, e.g. by leveraging flagship potential and helping identify threats. Despite scattered attempts to quantify birds’ aesthetic attractiveness to humans, there is no large-scale database providing homogeneous measures of aesthetic attractiveness that are comparable across bird species. We present data on the visual aesthetic attractiveness of bird species to humans, generated through an internet browser-based questionnaire. Respondents (n = 6,212) were asked to rate the appearance of bird species on a scale from 1 (low) to 10 (high) based on photographs from the Cornell Lab of Ornithology’s Macaulay Library. The rating scores were modeled to obtain final scores of visual aesthetic attractiveness for each bird. The data covers 11,319 bird species and subspecies, with respondents from multiple backgrounds providing over 400,000 scores. This is the first attempt to quantify the overall visual aesthetic attractiveness of the world’s bird species to humans.

## Background & Summary

Humans are fascinated by the diversity of forms and colors of wildlife species. Well-known examples throughout history include the festivals around the blooming of cherry trees in Japan^1^, interest in the flowers of orchids^2^ and the meanings of antlers of ungulates in hunting cultures^3^. Some of our all-time favorites among wildlife are birds. Ancient Egyptians imagined divinities as bird-headed gods, in the Roman Empire it was common to interpret omens from the behavior of birds. In modern societies birdwatching is an important touristic industry, while constantly emerging stories such as popular books and movies, or internet memes, give bird species cultural value^4–7^. Beyond these cultural values of wildlife, we are starting to appreciate that understanding the aesthetic attractiveness of species, including birds, is of importance also from a conservation standpoint. Amidst a global biodiversity crisis, shedding light on the factors that make us like a species or another can inform conservation actions, e.g., by helping us understand how humans perceive other species, leveraging flagship potential or helping us to identify potential threats. For instance, being a target for the legal and illegal trade of wildlife may be, in part, related to the visual aesthetic attractiveness of bird species^8–10^. Furthermore, evidence is accumulating that the charisma and physical, aesthetic attractiveness of species impacts the allocation of conservation funding and efforts^11–18^.

Aesthetic attractiveness is defined as a set of features that makes something interesting or likable to the observer and is usually composed of a combination of visual and emotional cues: Several methods have been used to understand the overall visual aesthetic attractiveness of other species to humans. Some studies have aimed at separating the visual traits which make each species attractive to humans^11,17,19^. Other studies have modelled the attractiveness of species through a combination of visuals, audio, and/or familiarity stimuli^8,9,12^. Also, it has been shown that in people’s perceptions an interplay of aesthetic and cultural values might be less important at more local than global scales^11^ yet the two types of values typically interact in some way in driving the perception of species: for instance locals might have a different perception than e.g. bird watching tourists^20,21^. Internet-based methods are increasingly popular to determine the amount or direction of human interest towards species^20,21^. However, these methods often measure the quantity of online mentions (the salience of a species on the internet)^6,22,23^. For example, relatively large attention to a societally important species might give it a lot of internet search hits and interest, but this only tells of one type of interest and does not mean e.g. that the species is visually more or less aesthetically attractive to humans^6,20,24^. Even if there are a multitude of cultural, emotional and value-related reasons behind people’s attitudes and perceptions of species^24–26^, it is of importance to understand also the amount of aesthetic attractiveness behind our perception of species.

Here, we present a data set on the visual aesthetic attractiveness of bird species to humans. The data are based on an internet browser-based questionnaire where respondents were asked to evaluate the appearance of bird species based on photographs. We asked respondents to give an overall rating (1 (low) – 10 (high)) of visual appearance of the bird depicted in the photo. We also gathered background information on the home country, demographics, birding skills, and nature-related attitudes of the respondents and used this information to generate visual aesthetic attractiveness score values for each species corrected for multiple confounding factors. By making global visual aesthetic attractiveness data for all bird species freely available, we open the possibility to answer multiple research questions on how birds’ visual aesthetic attractiveness to humans, in part, may affect our relationship to bird species, but also influence societal level aspects e.g. tourism, conservation investment, trade in bird species, and beyond.

## Methods

### The iratebirds application for rating birds’ appearance

**Fig. 1:**
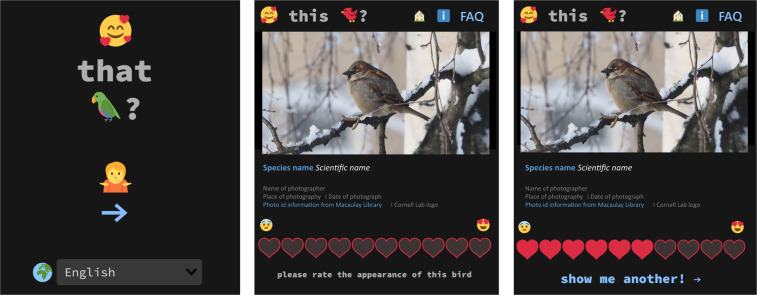
The user interface of the iratebirds application. The application was usable on internet browsers on all devices. The left panel shows the initial page where the user could select the preferred language, while the central and right panels show examples of the scoring pages where a bird photograph is available for rating (1 heart for least attractive to 10 for most attractive). The photograph on this figure is by Anna Haukka.

We collected the iratebirds data using an online survey in the format of an application. We designed the iratebirds.app -website to resemble globally familiar online applications (Fig. 1). On the website, the user first selected their user language (out of 20 options, see below) and was then directed to a page where they received information on the study, and gave consent to use their answer for research purposes. We did not collect any personal identification information, and we processed the data according to the privacy policy of the Finnish Biodiversity Information Facility (FinBIF)^27^. Upon starting the use of the app and after selecting the language, the user interface showed a photograph of a bird from the eBird’s Macaulay Library, a service owned by the Cornell Lab of Ornithology^28,29^ and a prompt to rate the looks of the bird (*“Please rate the appearance of this bird!”*) with hearts on a linear scale from 1 (low) to 10 (high). This scale was used to gain understanding on the amount of the visual aesthetic appeal of the pictured bird (Fig. 1). Users could not skip rating a bird. After rating a minimum of 10 birds, the user received a prompt to answer a background survey. After filling in the survey the person could choose to rate more bird species. Also, the respondent could choose to fill in the survey at a later stage via the menu on the iratebirds application.

We asked volunteers to translate the iratebirds application into 20 languages, which covered a wide range of different cultural regions. These languages were Arabic, Chinese, Dutch, English, Finnish, French, German, Hungarian, Italian, Indonesian, Japanese, Kannada (India), Korean, Latvian, Marathi (India), Portuguese (Portugal), Portuguese (South America), Russian, Spanish, and Swahili.

We distributed the application to as wide audiences as possible via social media (our own channels as well as collaborators channels on Facebook and Twitter), collaborators (e.g. research collaborators), email-lists (e.g. those of birding associations), a press-release in Finnish and English, and in Finland also via the radio and newspapers. Thus, the respondents are members of the public who have heard of the application via any of the mentioned means of communication. We timed the data collection between August 2020 and April 2021. The data set published here is based on all the ratings and survey replies collected until the 17^th^ April 2021.

### Using multiple photos per species to avoid the photograph’s quality impacting the ratings

For rating the birds, we used photographs from the eBird’s Macaulay Library, a service owned by the Cornell Lab of Ornithology^28,29^. The service has photographs of all living species and subspecies of birds. The photographs have a user-based quality-rating, which we used to choose the best photographs to be presented in the iratebirds application. The Macaulay Library has user guidelines for rating the quality of the photographs so that on a scale from 1 (low) – 5 (high) and therefore highly rated photographs should have the bird clearly visible and sharply shown on the photograph^30^. Specifically, we chose at random one of the five top user-rated photographs in the service per species. If less than five photos of a species existed in the database, we used all those available. This way the score for each bird species or subspecies was based on a sample of the best available photographs and the impact of the photographs’ quality on the ratings was minimized. For each user, we then randomized the order of bird species shown to minimize the impact of photo order on the ratings. Also, each user has seen a random sample of both local and exotic birds to them, diluting the familiarity effect of the species in the overall data.

The full library used for this study consisted of a total of 58,745 unique photographs, with an average of 5.7 (±1.2 s.d.) photos per bird species, and an average of 5.2 (±1.7 s.d.) photos per subspecies. The photographs used for rating can be viewed in the open access Macaulay Library Database^28^ by searching for them with the photographs’ ids (column ‘macaulay_photo_catalog_id’) provided in the raw data file ‘iratebirds_raw_data_taxonomy_photoinfo_ratings_survey_251022.csv’ which is found on Figshare^31^.

### Demographic information from the background survey

We asked the participants for consent to use their answers for research purposes (Table 1). The survey data gives background user data for 199,509 individual bird ratings. The answers provide information on self-stated bird identification skills, nature-related attitudes, being a professional in environmental or nature related fields, membership of birding or other environmental organization, as well as demographic information such as home country, age, and gender. The questions on nature and environment attitudes of the respondents were adapted from the “nature relatedness” (NR-6) - questionnaire^32^.

**Table 1:** Background survey questions on knowledge and relationship to birds and nature.

Question	Answer type	Column name in data set
**Questions on knowledge and relationship to birds**		
Are you able to identify the common birds found in your local area?	Likert-scale 1 (never) – 5 (every time)	identification_common_birds
Are you able to identify the rare birds found in your local area?	Likert-scale 1 (never) – 5 (every time)	identification_rare_birds
Is seeing birds an experience you find exciting and/or joyful?	Likert-scale 1 (never) – 5 (every time)	birds_bring_joy
Do you pay attention to birds wherever you go?	Likert-scale 1 (never) – 5 (all the time)	pays_attention_to_birds
Do you often spend time engaging with nature outdoors?	Likert-scale 1 (never) – 5 (very often)	spends_time_outdoors
**Questions on association and occupation**		
Do you belong to any local, national, or global birdwatching or ornithological associations (e.g., my local birding group, BirdLife, etc.,)?	Yes/No	bird_assoc_member
Are you a member of any local, national, or global, environmental or nature organisations that are not specifically focused on birdwatching or birds (e.g., Trust for Nature)?	Yes/No	env_org_member
Do you work in an environmental-/nature-based profession (e.g., biologist, nature tour-guide, natural resource manager, etc.)?	Yes/No	env_nature_professional
**Questions on location and demographic information**		
In which country do you currently live?	Open answer	respondent_home_country
In which year were you born?	Open answer	respondent_birth_year
What is your gender?	Open answer	respondent_gender

The questions cover nature and environment related attitudes, knowledge on birds, membership in nature or birding associations, professional background and demographic information on the home country and age of the person. Questions were asked on a Likert-scale, were of type yes/no or open ended.

### Data cleaning

**Fig. 2:**
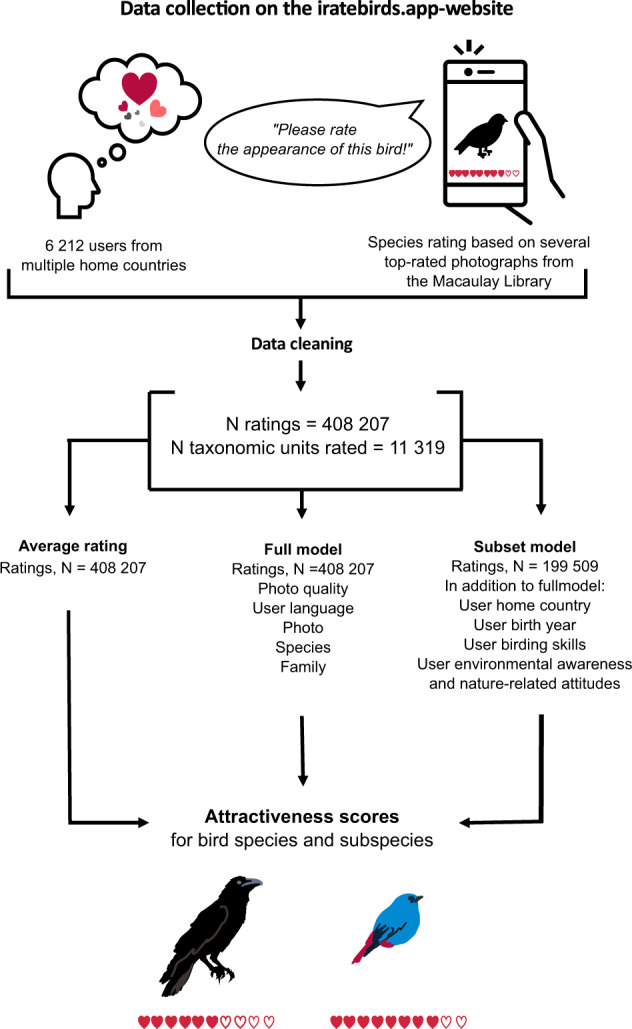
The steps of the iratebirds data collection and modelling. The figure shows how data were collected on the iratebirds application, then cleaned leaving 408,207 individual ratings for 11,319 taxonomic units. These data were used to calculate the average rating (n = 408,207 ratings), to create the full model (n = 408,207 ratings) and the fitted subset model (n = 199,509 ratings). These three different models each give attractiveness scores for bird taxonomic units at species, subspecies, and, in some cases, the species group level.

While cleaning the final data set (Fig. 2), we excluded records for two reasons:

1. For some rare or extinct species there were no good photographs available (e.g. only a photo of a museum-specimen or a book page with species information), or the photograph did not represent a bird.
2. We excluded duplicates of some ‘iratebirds_user_id’s that occurred more than once in the survey responses (e.g., several people using the app from the same device). Those replies have been excluded if they could not be reliably distinguished by different timestamps and different user survey responses. If we could reliably distinguish the replies and ratings by timestamps (e.g., a different date), and by a difference in the user survey replies, then the IDs were renamed with additions of “_2”, “_3”, etc. to be able to join the correct ratings and survey responses. There were only 17/6,212 impacted user ids, and the process thus created 17 new user ids.

Also, some records were modified or translated to have uniform data:

1. We translated the survey’s answers in all other languages back into English and replaced all other language metadata from Macaulay Library^28^ with English language metadata for the photographs (sex and age categories of the bird in each photograph).
2. We reclassified the gender category of the survey respondents from open answers into male, female, and non-binary.
3. We calculated the age of each respondent in the year when the iratebirds rating was done based on the birth year, and the age was added to the data set. We removed the birth year and age for two respondents who had not given a realistic response to this question.
4. Survey data is NA where the user did not participate in the survey.

The ‘iratebirds_user_id’ field was used to join the rating and survey data.

### Bird taxonomy

The data has an addition of taxonomic information based on the eBird/Clements integrated checklist v 2019^29^. The Macaulay Library^28^ used, at the time of data collection, this same taxonomy. We joined the full taxonomic data to the iratebirds ratings’ database based on the species or subspecies scientific names.

## Data Records

The iratebirds database is openly available in Figshare 10.6084/m9.figshare.20170082^31^. The database files are provided both as a comma delimited file (.csv) and as an Excel file (.xslx). Description of columns are in Tables 3, 4 in this manuscript and Supplement 1, Table S1, but also in the README file uploaded alongside the database in Figshare^31^. Figure 2 on this manuscript shows the steps from data collection to modelling.

The raw data files (iratebirds_raw_data_taxonomy_photoinfo_ratings_survey_251022.xlsx & iratebirds_raw_data_taxonomy_photoinfo_ratings_survey_251022.csv) include iratebirds.app ratings and respective survey responses from those app users who filled the survey as well as information on the photographs used for rating the appearance of the birds.

The modeled visual aesthetic attractiveness values database (iratebirds_final_predictions_average_fullmodel_subsetmodel_151122.xlsx & iratebirds_final_predictions_average_fullmodel_subsetmodel_151122.csv) includes visual aesthetic attractiveness of birds (N = 11,319), as perceived by humans, calculated in three different ways which one can consider for use, according to the specific research needs: i) raw average visual aesthetic attractiveness per species (or subspecies), ii) visual aesthetic attractiveness corrected for language group of the user and the quality of the photo used for scoring the birds appearance, iii) visual aesthetic attractiveness corrected as in ii) plus other user specific factors related to their relationship and knowledge on birds and nature, home country and other variables (Fig. 2). The latter represents a subset of all the bird species (N = 11,215). The data on visual aesthetic attractiveness scores are also available at the species level, and at the sex within species level, for the sexually dichromatic species (iratebirds_pred_ratings_species_and_sex_level_120123.xlsx & iratebirds_pred_ratings_species_and_sex_level_120123.csv).

On average, each user rated a total of 66 bird photographs and most of them some tens of photographs (range 1–3,588 ratings per application user id). Each species or subspecies received 1–65 ratings (average 36 ± 11 s.d.). Many users (1,754 out of 6,212 unique users) used the application in English, regardless of residing in another language zone, but most individual bird ratings were done by Finnish language users (1,884) (Table 2).

**Table 2:** Number of birds’ visual aesthetic attractiveness ratings and users from the iratebirds.app per language that was used in the user interface.

iratebirds user language	No of ratings	No of users
Finnish	178,344	1,884
English	78,699	1,754
Russian	43,770	650
Italian	31,396	542
Japanese	27,575	396
French	11,672	271
German	9,872	145
Latvian	8,191	129
Chinese	8,067	183
Spanish	7,511	199
Dutch	1,660	40
Hungarian	475	13
Marathi (India)	336	4
Portugese (Brazil)	305	17
Arabic	186	9
Portugese (Portugal)	148	4
TOTAL	408,207	6,240

The sum of users here is larger (6,240) than the total number of unique users (6,212) as the user id was device based. 28 user ids have been giving responses in two different languages as it has been possible to switch user language on the same device even during one rating session.

Overall, 2,785 users out of 6,212 filled in the background survey (questions shown in Table 1), and for them we also have demographic information, which is missing for the rest of the respondents and their bird ratings. Tables S3–S10 in Supplement 1 show the number of bird ratings for each survey response category, giving demographic and background information for the users. In FigShare^31^, there is an additional file showing the number of ratings for each user id, together with their demographic information, for those who filled the survey (count_ratings_per_user_demographics.xlsx & count_ratings_per_user_demographics.csv).

## Technical Validation

### Estimation of attractiveness scores

**Fig. 3:**
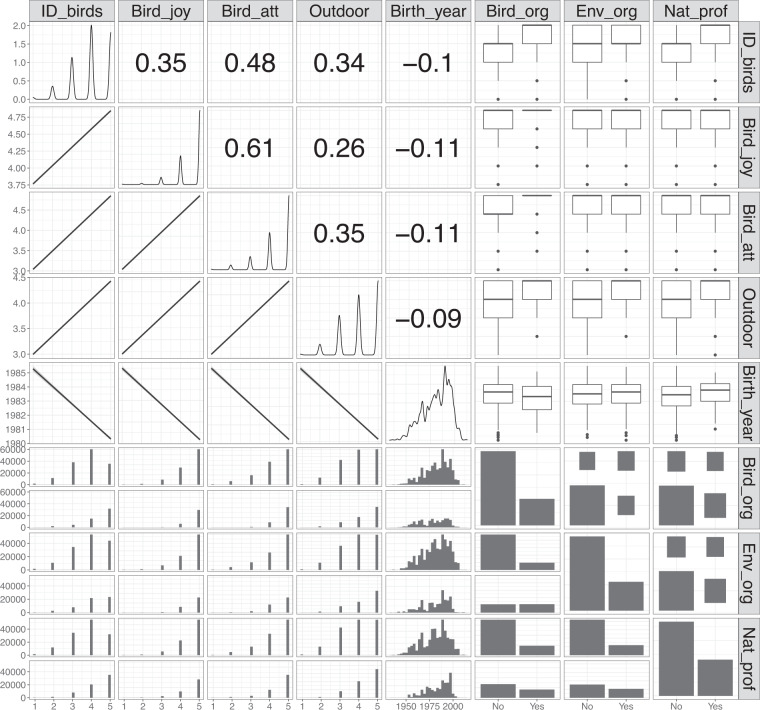
Multicollinearity assessment (generalized pair plot) among continuous and categorical predictors included in the regression models. On the diagonal, variable distribution. Above the diagonal: Pearson’s r correlations (all below the threshold of 0.7 by Zuur *et al*.^36^) for continuous variables, and association plots for categorical vs continuous and categorical vs categorical. Below the diagonal are association plots among predictors. Variable names have been shortened for the figure and the full variables names in the data set are: ID_birds = identification_common_birds, Bird_joy = birds_bring_joy, Bird_att = pays_attention_to_birds, Outdoor = spends_time_outdoors, Birth_year = respondent_birth_year, Bird_org = bird_assoc_member, Env_org = env_org_member, Nat_prof = env_nature_professional.

We performed all analyses and calculations in R version 4.1.0^33^. We calculated consensus visual aesthetic attractiveness scores for each taxonomic unit (species or subspecies) using three indexes. The aim was to get an overall average visual aesthetic attractiveness score for the bird species and subspecies over all raters of the birds’ appearance. We generated a consensus score as the average individual raw data scores for each taxonomic unit. Next, we calculated the predicted score for each taxonomic unit conditional of the data we had. In doing so, we fitted two regression models designed to predict the score of each taxonomic unit while controlling for confounding factors related to taxonomy (e.g. bird family), attributes of the rater (e.g. language), and attributes of the photo (e.g. the quality of the rated photo). Since the dependent variable is an ordinal score from 0 to 9 (transformed from original scores 1–10), we fitted regressions for ordinal data within a Bayesian framework using the R package ‘brms’^34,35^. Specifically, we set a cumulative error family and a logit link function with equidistant threshold among the ordered scores and default settings (four chains, 2,000 iterations, and 1,000 iterations as burn-in). For all models, prior to model fitting, we followed the general protocol of Zuur *et al*. (2010) for data exploration^36^. Figure 3 shows that there is no collinearity with the variables used. We validated all models by inspecting mixing of the four chains with the function *mcmc_plot* in brms (no divergence was detected).

We fitted the first regression model (hereafter ‘full model’) including all confounding factors that we collected for all users rating birds directly in the web app (n = 408,207 individual ratings; mean ± s.d. number of ratings per taxonomic unit: 35.1 ± 11.8) (Fig. 2). The structure of the full model (in R notation of brms function) was:

rating ~ photograph’s quality + (1|bird species) + (1| user language) + (1| photograph) + (1|bird family)

In the code and database, the variables are named: photograph’s quality = macaulay_photo_user_rating_may21, bird species = sci_name, user language = iratebirds_app_language, photograph = macaulay_photo_catalog_id, bird family = family.

In the above model we controlled for the quality of the photo (macaulay_photo_user_rating_may21) as a continuous covariate, and we included four random intercept factors that accounted for pseudoreplication for each species (sci_name) and photo (macaulay_photo_catalog_id) and for the potential confounding effect of users’ language (iratebirds_app_language) and phylogenetic relationship of species within the same family (family). In other words, one may expect that species within the same family share more similar attractiveness scores than expected from random (Fig. 3). Similarly, rating from users within the same language may give more similar scores than expected from random due to a common cultural background.

We fitted the second model (hereafter ‘subset model’) with all confounding factors above, as in the ‘full model’ plus eight additional confounding factors referring to users’ affinity with birdwatching and ornithology as well as their general environmental awareness. All these information was obtained from a questionnaire filled by a subset of users (see section “Demographic information from the background survey”); hence, this subset model had a reduced sample size compared to the full model [n = 198,537 individual ratings; mean ( ± s.d.) number of ratings per taxonomic unit: 17.2 ± 6.4]. The structure of the subset model (in R notation of brms function) was:

rating ~ photograph’s quality + respondents bird identification skills + happiness of seeing birds + bird noticing skills + outdoor habits + membership of birding association + membership of environmental organisation + environment or nature professional + respondent age + (1|bird species) + (1|respondent home country) + (1| user language) + (1| photograph) + (1|bird family).

In the code and database the variables are named: photograph’s quality = macaulay_photo_user_rating_may21, respondent’s bird identification skills = identification_common_birds, happiness in seeing birds = birds_bring_joy, bird noticing skills = pays_attention_to_birds, outdoor habits = spends time outdoor, membership of birding association = bird_assoc_member, membership of environmental organization = env_org_member, environment or nature professional = env_nature_professional, respondent age = respondent_birth_year, respondent home country = respondent_home_country, bird species = sci_name, user language = iratebirds_app_language, photograph = macaulay_photo_catalog_id, bird family = family.

In this subset model we controlled for all previously discussed confounding factors (macaulay_photo_user_rating_may21, sci_name, macaulay_photo_catalog_id, and family), and with home country replacing user language. This was done to ensure that a higher resolution of spatial data regarding the user was used, as home country (respondent_home_country) had much higher levels of detail compared to main language, e.g. English, or French, which could apply to many countries in the world. In this subset model, several user-specific variables were included as continuous and categorical covariates. These referred to users’ demography (birth_year), experience with birdwatching and ornithology (identification_common_birds, birds_bring_joy, pays_attention_to_birds, bird_assoc_member), and general environment awareness and nature-related attitudes (spends_time_outdoors, env_org_member, env_nature_professional env_org_member, env_nature_professional). Table 1. includes a list of the user survey questions used to create these variables.

**Fig. 4:**
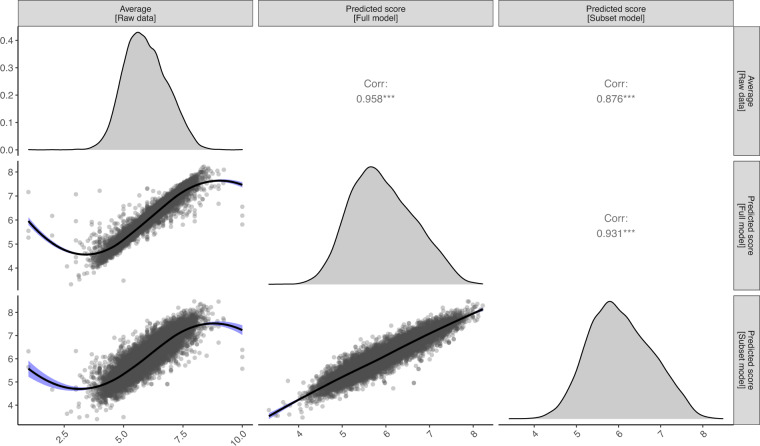
Pairwise Pearson’s correlations among the three indices of attractiveness (average, predicted value from the full model, and predicted value of the subset model) calculated for each taxonomic unit (n = 11,319). Density plots on the diagonal display the distribution of values (the top-most y-axis showing the scale for these plots), bivariate scatter plots are displayed below the diagonal (the two lower y-axis scales apply to these), and the Pearson r correlations above the diagonal. For each scatter plot, regression lines are included for visual presentation.

**Fig. 5:**
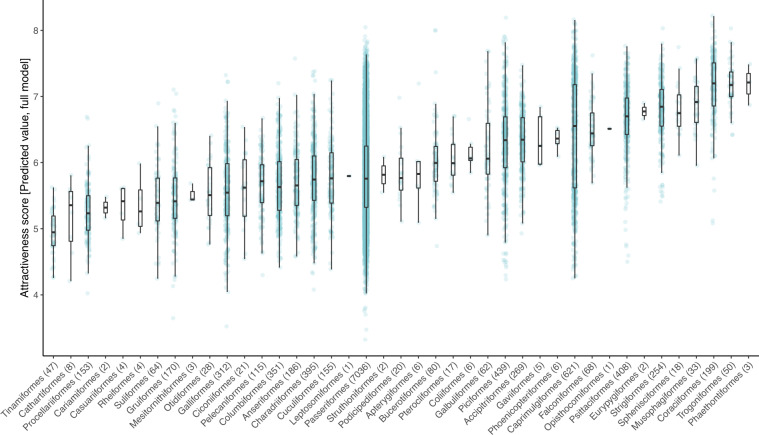
The average and dispersion of attractiveness scores per taxonomic order of birds (in ascending order of the mean attractiveness), in the full model. The numbers on the x-axis show the number of species in the order of birds.

We used the above two models (full and subset model) to predict the score for each individual rating, thereby obtaining scores corrected for the confounding factors above. To generate these predictions, we first obtained the predicted probability for each level of the ordinal score (i.e., from 1 to 10). Next, for each row of the database, we draw 1,000 random levels, weighted by the predicted probabilities, and took the average of the 1,000 random scores. Finally, we averaged all the predicted scores separately for each taxonomic unit to generate a final consensus attractiveness value. For all the three indices of attractiveness (raw average, predicted score from full model and from subset model), as a measure of uncertainty, we also report the standard deviation of individual scores for each taxonomic unit, and show the number of photos and ratings used to derive each score (Table 3). Figure 4 shows pairwise Pearson’s *r* correlations among the three attractiveness indices. Figure 5 shows the average and dispersion of attractiveness scores per order of birds, based on the full model.

**Table 3:** Description of each column in the modeled species/subspecies rating scores’ database.

Variable	Description
***eBird/Clements integrated checklist v. 2019 data columns***	
order	scientific name at the bird order level
family	scientific name at the bird family level
ebird_species_group	English language species group as defined in the eBird taxonomy
sci_name	scientific name of the bird species or subspecies or species group
common_name	bird species or subspecies English language name
species_category	species categories as listed in the eBird/Clements checklist: species, form, group (monotypic), group (polytypic), domestic
***iratebirds ratings data columns***	
average_attractiveness_rawdata	the average visual aesthetic attractiveness score of the species/subspecies/species group, calculated from the raw iratebirds rating data
sd_rawdata	the standard deviation of the average visual aesthetic attractiveness score
predicted_attractiveness_full_model	the average visual aesthetic attractiveness of the species/subspecies/species group, from the full model
sd_full_model	the standard deviation of the full model’s visual aesthetic attractiveness score
predicted_attractiveness_subset_model	the average visual aesthetic attractiveness of the species/subspecies/species group, from the subset model
sd_subset_model	the standard deviation of the subset model’s visual aesthetic attractiveness score
no_of_photos_used	the number of photographs rated in the iratebirds app, for the bird taxa in question
no_of_ratings_used	the number of ratings given in the iratebirds app, for the bird taxa in question

The data include information on the taxonomy of the bird species, based on the eBird/Clements integrated checklist v. 2019, and the average, full model, and subset model values.

### Visual aesthetic attractiveness score for sexually dichromatic species

Many bird species show strong sexual dichromatism, whereby one of the sexes, often the male, expresses more colorful and extravagant plumage^37^. Consequently, we quantified attractiveness scores using the same approach as the full model described above, but now focusing on the species (not subspecies) and sex level (whereby 31,181 ratings from 2,915 species were given for photographs of birds classified as males, and 8,827 ratings from 1,166 species of female birds; the rest had unknown sex). Specifically, we obtained the species level name for each Macaulay Library^28^ photograph rating by isolating the binomial name from the full name given by the eBird/Clements integrated checklist v. 2019^38^ that also includes subspecies. We then used the information from the Macaulay library photographs’ metadata whereby users also assign the sex of the subject in the photograph, for those species whose dichromatism allows such sex distinction. This gender variable from Macaulay Library^28^ thus included three levels: male, female, and unknown sex. We then added the species and sex level (“species_sex”) as a random intercept of a model that closely followed the one described above for the full dataset analyses (in R notation of 'brms' function):

attractiveness rating ~ photograph’s quality + (1|bird species and sex) + (1|user language) + (1| photograph) + (1|bird family)

In the code and database, the variables are named: photograph’s quality = macaulay_photo_user_rating_may21, bird species/sex = sci_name_sex, user language = iratebirds_app_language, photograph = macaulay_photo_catalog_id, bird family = family.

In this model, the additional “sci_name_species_sex” random intercept captures the sex level within a given sex dichromatic species (e.g. *Aceros nipalensis_f*, *Aceros nipalensis_m*, *Aceros nipalensis_u* indicate that the photo referred to a female, male or unknown sex of the rufous-necked hornbill, respectively).

We then applied the same prediction as detailed above as the ‘full model’ for obtaining the predicted attractiveness score by species and sex. The visual aesthetic attractiveness scores from this model are given in files iratebirds_pred_ratings_species_and_sex_level_120123.xlsx & iratebirds_pred_ratings_species_and_sex_level_120123.csv in the data repository in Figshare^31^. Description of the variables is in Table 4. below, as well as in the README-file given together with the data on Figshare^31^.

**Table 4:** Description of each column in the modeled species ratings scores database, where separate scores are given for male and female individuals.

Variable	Description
number	number of the row in the database
***eBird/Clements integrated checklist v. 2019 data columns***	
sci_name	scientific name of the bird species, according to eBird/Clements checklist
*Macaulay Library data columns*	
sex	sex of the bird on the photo, male/female/unknown
***iratebirds ratings data columns***	
predicted_attractiveness_sex_model	The average modelled visual aesthetic attractiveness rating for the species at the sex level
sd_sex_model	The standard deviation for the modelled average visual aesthetic attractiveness score for the species at the sex level

The data include the scientific name of the bird species and the modeled ratings for the male, female, and unknown sex representatives of the bird species. These data can be used to have distinct scores of attractiveness in the case of sexually dichromatic bird species.

## Usage Notes

Here, we have generated data for the overall scores of visual aesthetic attractiveness of 11,319 bird taxonomic units based on an internet application. The iratebirds data for the visual aesthetic attractiveness of bird species to humans can be used for several research purposes, bearing in mind certain limitations of the dataset. Understanding birds’ aesthetic attractiveness can be, in part, used to explore human-bird relationships and e.g. direct conservation marketing and to understand which are useful flagship bird species.

One potential further step could be to investigate, on a large scale, if humans generally have only one set of phenotypic traits that they prefer in birds or if there are different combinations of traits which make them aesthetically attractive to people. Therefore, it would be useful to examine which phenotypic traits of the birds, such as color or size, drive their visual aesthetic attractiveness to humans. This can, in part, and keeping in mind that there are other drivers of attractiveness too, be used to understand e.g., which traits drive the trade of bird species, people’s attitudes towards birds or conservation funding. In doing such research, it is good to bear in mind that the iratebirds data is a large-scale model and in more localized contexts or specific cultural settings the overall attractiveness from our data set should be used with caution. For instance, it has been previously shown that a local study might give information on how, for instance, different groups of people, or people who live in different locations, have different values, attitudes or attractiveness for the same bird species^21,39,40^. Therefore, our data cannot always be used to draw the straight-forward conclusion that the most attractive birds for individual people or certain groups of people, would always be the highest scoring ones from the iratebirds data set. However, as we publish also the raw data from the application, it is possible to use only a subset of our data at the more local contexts where our data set has sufficient replies, e.g. by filtering scores coming from respondents in a specific country or otherwise defined cultural region.

As a conclusion of the previous, it might be worth comparing our results on bird attractiveness to results of other such studies based on different methods (even for a subset of species if global coverage of species is not available in other data sets). This would allow to create understanding on whether local perceptions might differ from the average global perceptions on birds’ aesthetic value to people, and what are the drivers of those differences. Also, an addition of birds’ sounds’ aesthetic appeal would be informative to also understand the vocal aspects of birds’ aesthetics to humans. Aesthetic attractiveness can be used to direct conservation marketing and to understand which are useful flagship bird species. In addition to this, conservation culturomic studies^41^, where large scale digital data sets are used to understand e.g. human’s perception on different nature related topics, can benefit from this dataset. It can be used together with other data to monitor the overall cultural interest of bird species including aesthetic value.

Aesthetic attractiveness values can give insights to the way people value and have interest towards bird species. A similar study on attractiveness could easily be repeated for other taxa, given that a comprehensive set of photos is available to use. Also, this study on birds does not cover, in a balanced way, all the cultures of the world, and therefore it would be useful to further extend or repeat the study to collect even more ratings e.g., from the cultures that we did not cover.

As a concluding remark, as we publish the raw data from the iratebirds application and user survey we do also point out that it is possible to build other models with it besides the one we publish here .

### Usage limitations

Users should take note that the data set does not cover all cultures globally even though it is representative of respondents from several cultural backgrounds (Table S2 in Supplement 1). Also, we have not, for instance, compared the responses of people from different backgrounds, but have aimed to have the average overall visual aesthetic attractiveness rating across demographies. However, our hierarchical models show that the demographic background, birding skills, or nature-related attitudes of the respondents does not, at large, impact the average attractiveness scoring of the bird species (Fig. 3) and therefore the use of the full model’s scores in our dataset can give reliable average insights into the visual aesthetic attractiveness of birds to people. Also, the full model has been corrected for certain biases such as lower quality of photos or low number of raters per bird and is therefore a better score than the raw average from the ratings.

When choosing to use only a section of the data, the user might want to keep in mind the following: We do not have demographic user data for all the ratings, so if that is important to consider, select only those ratings for which it is given (the raw data file ‘iratebirds_raw_data_taxonomy_photoinfo_ratings_survey_251022.csv’ has a column called ‘survey_consent’ in which the value ‘Yes’ is given for the users who provided demographic data). Also, it’s worth checking how many users rated the bird species and on how many photos the score is based on so that the photograph’s quality of one picture does not impact the rating in cases where only one photograph was used. There are 188 cases (1.7% of total bird species) where we had only one photo per bird used for ratings, and 39 out of those (0.03% out of total bird species) have been rated by only one user.

It might be, for the user of this data, important to check where the users of the subset of data come from, is the subset culturally representative? From the raw data file (iratebirds_raw_data_taxonomy_photoinfo_ratings_survey_251022.csv’) it is possible to do a selection of bird species that live only in a certain region or to select only ratings from users who are of certain demographics, e.g., by age or home country.

The taxonomy of this data follows the eBird/Clements integrated checklist v. 2019^38^. It is recommended to update and/or match the taxonomy before use or before joining with other bird species data sets.

## Supplementary information

Supplement 1

## Data Availability

The ‘iratebirds.app’ application code can be found on GitHub https://github.com/luomus/iratebirds^42^. R code used to generate the modeled ratings (Haukka_et_al_iratebirds_Scientific_Data_Data_Modelling.R) as well as figures (Haukka_et_al_iratebirds_Scientific_Data_Figure.R) and tables (Haukka_et_al_iratebirds_Scientific_Data_Tables.R) in this manuscript and the supplementary file are available in Figshare alongside the data set^31^.
