# Supplementary material for: The iratebirds Citizen Science Project: a Dataset on Birds’ Visual Aesthetic Attractiveness to Humans
Source: Sci Data. 2023 May 19;10:297. doi: 10.1038/s41597-023-02169-0 (PMC10198970; doi:10.1038/s41597-023-02169-0)
Supplement: Supplementary file 1 — Supplement 1 [file 41597_2023_2169_MOESM1_ESM.pdf]

Supplementary information for

Haukka A, Lehikoinen A, Mammola S, Morris W, Santangeli A, 2023. The iratebirds -citizen science project: A dataset of birds' visual aesthetic attractiveness to humans, Scientific Data.

Dataset DOI: 10.6084/m9.figshare.20170082

Supplementary materials table of contents

SUPPLEMENTARY TABLES

Table S1: Description of each column in the raw data

Table S2: Number of birds' visual aesthetic attractiveness ratings and users from the iratebirds application per user country, in descending order of the number of ratings.

Table S3: Number of birds' visual aesthetic attractiveness ratings and users from the iratebirds application per gender.

Table S4: Number of birds' visual aesthetic attractiveness ratings and users from the iratebirds application per respondent age group.

Table S5: Number of birds' visual aesthetic attractiveness ratings from the iratebirds application per replies to Likert scale 1–5 questions on bird identification skills and the joy of seeing birds.

Table S6: Number of users from the iratebirds application per replies to Likert scale 1–5 questions on bird identification skills and the joy of seeing birds.

Table S7: Number of bird visual aesthetic attractiveness ratings from the iratebirds application per replies to questions on being an environment or nature professional and membership of birding or environmental organisations.

Table S8: Number of users from the iratebirds application per reply to questions on being an environment or nature professional and membership of birding or environmental organisations.

Table S9: Number of birds' visual aesthetic attractiveness ratings and users from the iratebirds application per replies to Likert scale 1–5 questions on time spent engaging with nature outdoors.

Table S10: Number of bird visual aesthetic attractiveness ratings, and the users giving each answer, from the iratebirds application for answers to Likert scale 1–5 question on how often the respondent pays attention to birds where ever one goes.

Table S1. Description of each column in the raw data. This data includes taxonomic and eBird database information on each bird species/species group/subspecies, data on the photographs used for visual aesthetic attractiveness rating as well as the original ratings records. The last section includes user replies to the background survey's questions (the questions are on Table 1 in the main manuscript).

Variable	Description
rating_id	running number of the rows, each row represents one rating score from the iratebirds application
eBird/Clements integrated checklist v. 2019 data columns	
order	scientific name at the bird order level
family	scientific name at the bird family level
ebird_species_group	English language species group as defined in the eBird taxonomy
sci_name	scientific name of the bird species or subspecies or species group
sci_name_species	scientific name at the bird species level (no group names or subspecies names)
sci_name_species_sex	scientific name at the bird species level with additional information on the sex of the bird on the photo, m = male, f = female, u = unknown
common_name	bird species or subspecies English language name
species_category	species categories as listed in the eBird/Clements checklist: species, form, group (monotypic), group (polytypic), domestic
ebird_species_code	eBird's own species ID, some subspecies might be under the same code as the species
ebird_taxon_order	running number of the taxon in the eBird/Clements checklist
ebird_checklist_id	taxonomy ID on the eBird/Clements checklist
extinct	species extinct = 1, not extinct = NA
extinct_year	estimated year of extinction
species_range	description of the habitat type and geographic location where the species is present
iratebirds.app rating data columns (uploaded on 17th April 2021), incl. Macaulay Library data on the rated photograph	
sex_bird_photo	the sex of the bird on the photo: male/female/unknown
age_bird_photo	the age of the bird on the photo: adult/juvenile/NA
macaulay_photo_catalog_id	the Macaulay Library catalog ID of the photo, can be used to search the rated photo from the Macaulay Library
macaulay_photographer_user_id	Macaulay Library/eBird user ID of the person who submitted the photo (public information on the Macaulay Library)
photo_latitude	coordinate location of photo, latitude coordinate as submitted by the photographer
photo_longitude	coordinate location of photo, longitude coordinate as submitted by the photographer
macaulay_rating_count_may2021	The count of user ratings for the photo on Macaulay Library in May 2021 (at the time it was used in the iratebirds app), Macaulay library guidelines for user ratings can be found at: https://support.ebird.org/en/support/solutions/articles/48001064480-rating-media (accessed 01.11.2022)
macaulay_photo_user_rating_may2021	quality rating of the photo on Macaulay Library website in May 2021. Macaulay Library's guidelines for user ratings can be found at: https://support.ebird.org/en/support/solutions/articles/48001064480-rating-media (accessed 01.11.2022)
photo_width_px	photo size measure, width in pixels
photo_height_px	photo size measure, height in pixels

Variable	Description
source	photo source: ebird = eBird or ml = Macaulay Library
iratebirds_user_id	individual user ID created by the iratebirds.app website, device based, was used to connect survey answers to the ratings
iratebirds_rating	rating 1-10 given by the user of the iratebirds.app to the bird on the photograph
iratebirds_app_language	language in which the iratebirds.app was used
iratebirds_timestamp	timestamp of the rating done on the iratebirds.app
iratebirds user survey/questionnaire data columns (uploaded on 17th April 2021)	
survey_language	language in which the demographic survey was filled by the user
survey_timestamp	timestamp of filling the demographic survey
survey_consent	yes/no answer to user giving consent to use the survey data for research purposes
identification_common_birds	1 (never)- 5 (every time) Likert scale answer to question "Are you able to identify the common birds found in your local area?"
identification_rare_birds	1 (never)- 5 (every time) Likert scale answer to question "Are you able to identify the rare birds found in your local area?"
birds_bring_joy	1 (never)- 5 (every time) Likert scale answer to question "Is seeing birds an experience you find exciting and/or joyful?"
pays_attention_to_birds	1 (never)- 5 (all the time) Likert scale answer to question "Do you pay attention to birds wherever you go?"
spends_time_outdoors	1 (never)- 5 (very often) Likert scale answer to question "Do you often spend time engaging with nature outdoors?"
bird_assoc_member	yes/no answer to question "Do you belong to any local, national, or global birdwatching or ornithological associations (e.g., my local birding group, BirdLife, etc.,)?"
env_org_member	yes/no answer to question "Are you a member of any local, national or global, environmental or nature organisations that are not specifically focused on birdwatching or birds (e.g., Trust for Nature)?"
env_nature_professional	yes/no answer to question "Do you work in an environmental-/nature-based profession (e.g., biologist, nature tour-guide, natural resource manager, etc.)?"
respondent_home_country	home country of user, open field answers (edited and translated into English)
respondent_birth_year	birth year, open field answers
respondent_age_in2020	respondent age at the time of data collection (calculated based on birth year)
respondent_gender	gender, open field answers, classified into categories male/female/binary/NA

Table S2. Number of birds’ visual aesthetic attractiveness ratings and users from the iratebirds application per user country, in descending order of the number of ratings. The replies have been given by respondents who answered to the voluntary user survey.

Home country	No of ratings	No of users
No home country available	197,646	3,474
Home country available	210,561	2,738
Finland	94,484	910
Russia	28,434	353
Italy	15,293	307
Japan	14,804	169

Home country	No of ratings	No of users
Germany	5,826	71
Australia	5,195	79
United Kingdom	4,210	80
France	4,146	101
Spain	4,035	86
Latvia	3,774	71
Belgium	3,614	62
Croatia	1,153	17
Austria	1,092	19
Ukraine	976	20
Macedonia	959	7
Netherlands	885	20
Estonia	876	12
Belarus	748	5
Switzerland	720	12
Canada	660	16
Ecuador	613	2
Bulgaria	607	8
Nigeria	606	9
Poland	584	6
Colombia	569	11
India	532	15
Sweden	527	17
South Africa	509	9
Portugal	357	6
Hungary	329	4
Ireland	319	2
Norway	310	10
Madagascar	285	1
Thailand	245	1
United Arab Emirates	238	1
Kenya	227	2
New Zealand	223	3
Tanzania	199	3
Brazil	186	10
Argentina	134	4
Malta	127	3
Seychelles	117	2
Montenegro	112	3
Turkey	103	3
Greece	101	2
Uzbekistan	97	4
Peru	85	1
Taiwan	84	5
Hong Kong	83	1

Home country	No of ratings	No of users
Denmark	74	3
Indonesia	72	5
Malaysia	59	1
Tunis	53	1
China	52	3
Mexico	46	4
Albania	44	1
Israel	43	2
Singapore	42	3
Czech Republic	35	1
Senegal	34	1
Luxembourg	33	2
Chile	31	2
Lebanon	30	1
Cyprus	28	2
Iceland	28	1
The Democratic Republic of Congo	18	1
Oman	17	2
Armenia	17	1
Serbia	17	1
Philippines	14	1
Saudi Arabia	14	1
Mallorca	12	1
Ghana	11	1
Romania	11	1
Andorra	10	1
Bolivia	10	1
Guinea-Bissau	10	1
Slovenia	10	1

Table S3. Number of birds' visual aesthetic attractiveness ratings and users from the iratebirds application per gender. The replies have been given by respondents who answered to the voluntary user survey. Replies saying 'unknown' were interpreted as the gender information not being available.

Gender	No of ratings	No of users
Gender not available	221,597	3,574
Gender available	202,866	2,380
Female	148,981	1,735
Male	35,450	614
Non-binary	2,179	31

Table S4. Number of birds' visual aesthetic attractiveness ratings and users from the iratebirds application per respondent age group. The replies have been given by respondents who answered to the voluntary user survey.

Age in 2020 by age group	No of ratings	No of users
No age information	207,379	3,565
Age information exists	200,828	2,647
80–89	168	5
70–79	2,959	42
60–69	15,857	209
50–59	26,513	334
40–49	40,800	483
30–39	52,460	659
20–29	54,715	806
7–19	7,356	109

Table S5. Number of birds' visual aesthetic attractiveness ratings from the iratebirds application per replies to Likert scale 1–5 questions on bird identification skills and the joy of seeing birds. The replies have been given by respondents who answered to the voluntary user survey.

	Number of ratings with response		
Likert scale answers	Identification of common birds in local area	Identification of rare birds in local area	Seeing birds is an experience I find exiting and/or joyful
Answer not available	195,173	196,240	195,510
Answer available	213,034	211,967	212,697
Never 1	2,066	24,986	626
2	14,437	53,818	2,297
3	44,958	64,789	13,785
4	77,906	51,695	58,224
Every time 5	73,667	16,679	137,765

Table S6. Number of users from the iratebirds application per replies to Likert scale 1–5 questions on bird identification skills and the joy of seeing birds. The replies have been given by respondents who answered to the voluntary user survey.

	Number of users with response		
Likert scale answers	Identification of common birds in local area	Identification of rare birds in local area	Seeing birds is an experience I find exiting and/or joyful
Answer not available	3,432	3,443	3,434
Answer available	2,780	2,769	2,778
Never 1	65	418	14
2	252	760	73
3	564	696	213
4	990	633	659
Every time 5	909	262	1,819

Table S7. Number of bird visual aesthetic attractiveness ratings from the iratebirds application per replies to questions on being an environment or nature professional and membership of birding or environmental organisations. The replies have been given by respondents who answered to the voluntary user survey.

	Number of ratings with response		
Are you a/an...?	Environment or nature professional	Member of a birding association	Member of another environmental organisation
Answer not available	195,075	195,592	195,140
Answer available	213,132	212,615	213,067
No	144,257	157,354	154,703
Yes	68,875	55,261	58,364

Table S8. Number of users from the iratebirds application per reply to questions on being an environment or nature professional and membership of birding or environmental organisations. The replies have been given by respondents who answered to the voluntary user survey.

	Number of users with response		
Are you a/an...?	Environment or nature professional	Member of a birding association	Member of another environmental organisation
Answer not available	3,429	3,434	3,433
Answer available	2,783	2,778	2,779
No	1,760	1,954	1,984
Yes	1,023	824	795

Table S9. Number of birds' visual aesthetic attractiveness ratings and users from the iratebirds application per replies to Likert scale 1–5 questions on time spent engaging with nature outdoors. The replies have been given by respondents who answered to the voluntary user survey.

Likert scale answers	Time spent engaging with nature outdoors, number of ratings	Time spent engaging with nature outdoors, number of users
Answer not available	195,021	3,428
Answer available	213,186	2,784
Never 1	985	19
2	11,692	172
3	44,015	524
4	69,038	924
Very often 5	87,456	1,145

Table S10. Number of bird visual aesthetic attractiveness ratings, and the users giving each answer, from the iratebirds application for answers to Likert scale 1–5 question on how often the respondent pays attention to birds where ever one goes. The replies have been given by respondents who answered to the voluntary user survey.

Likert scale answers	Paying attention to birds wherever one goes, number of ratings	Paying attention to birds wherever one goes, number of users
Answer not available	195,427	3,435
Answer available	212,780	2,777
Never 1	953	29
2	7,490	149
3	21,462	340
4	60,752	743
All the time 5	122,123	1,516
